# Parkinson's Disease Subtypes Identified from Cluster Analysis of Motor and Non-motor Symptoms

**DOI:** 10.3389/fnagi.2017.00301

**Published:** 2017-09-20

**Authors:** Jesse Mu, Kallol R. Chaudhuri, Concha Bielza, Jesus de Pedro-Cuesta, Pedro Larrañaga, Pablo Martinez-Martin

**Affiliations:** ^1^Department of Computer Science, Boston College Chestnut Hill, MA, United States; ^2^Department of Basic and Clinical Neuroscience, The Maurice Wohl Clinical Neuroscience Institute, King's College London London, United Kingdom; ^3^Computational Intelligence Group, Department of Artificial Intelligence, Universidad Politécnica de Madrid Madrid, Spain; ^4^National Center of Epidemiology, Instituto de Salud Carlos III Madrid, Spain

**Keywords:** Parkinson's disease, subtypes, non-motor symptoms, motor symptoms, cluster analysis

## Abstract

Parkinson's disease is now considered a complex, multi-peptide, central, and peripheral nervous system disorder with considerable clinical heterogeneity. Non-motor symptoms play a key role in the trajectory of Parkinson's disease, from prodromal premotor to end stages. To understand the clinical heterogeneity of Parkinson's disease, this study used cluster analysis to search for subtypes from a large, multi-center, international, and well-characterized cohort of Parkinson's disease patients across all motor stages, using a combination of cardinal motor features (bradykinesia, rigidity, tremor, axial signs) and, for the first time, specific validated rater-based non-motor symptom scales. Two independent international cohort studies were used: (a) the validation study of the Non-Motor Symptoms Scale (*n* = 411) and (b) baseline data from the global Non-Motor International Longitudinal Study (*n* = 540). *k*-means cluster analyses were performed on the non-motor and motor domains (domains clustering) and the 30 individual non-motor symptoms alone (symptoms clustering), and hierarchical agglomerative clustering was performed to group symptoms together. Four clusters are identified from the domains clustering supporting previous studies: mild, non-motor dominant, motor-dominant, and severe. In addition, six new smaller clusters are identified from the symptoms clustering, each characterized by clinically-relevant non-motor symptoms. The clusters identified in this study present statistical confirmation of the increasingly important role of non-motor symptoms (NMS) in Parkinson's disease heterogeneity and take steps toward subtype-specific treatment packages.

## Introduction

Parkinson's disease (PD) is classically considered a motor disorder, with resting tremor, rigidity, bradykinesia, and postural instability and gait disorder as its core features. However, the concept of PD has changed considerably in the last few years, now prompting a revision of its diagnostic criteria to include non-motor symptoms (NMS) in the core parameters (Postuma et al., 2015; Marras and Chaudhuri, 2016). There has been growing recognition that NMS in PD are caused by neurotransmitter pathway dysfunctions which involve both the central and peripheral nervous systems (Jellinger, 2012; Gjerløff et al., 2015). The significant clinical heterogeneity of NMS in PD suggests the existence of specific non-motor subtypes (Marras and Chaudhuri, 2016; Sauerbier et al., 2016).

Previous cluster analyses have already identified motor- and non-motor-based clusters in PD patients (e.g., van Rooden et al., 2011; Erro et al., 2013; Ma et al., 2015; Pont-Sunyer et al., 2015). Recently, it has been argued that the recent concept of non-motor endophenotypes of PD provides a stronger basis for subtyping, since these relate to the central pathophysiology of specific neurotransmitter systems and are therefore likely to remain stable over time (Marras and Chaudhuri, 2016). As such, several studies have explored PD subtypes while considering motor subtypes and their association with non-motor aspects of the disease such as, psychopathology and cognition (Graham and Sagar, 1999; Reijnders et al., 2009; Selikhova et al., 2009; Burn et al., 2012; Flensborg Damholdt et al., 2012), REM sleep behavior disorder (Romenets et al., 2012), and daily visual activities (Seichepine et al., 2011). To our knowledge, however, no studies have used cluster analysis techniques to examine subtypes present in NMS only.

In this study, we used cluster analysis techniques to search for PD subtypes from a large, multi-center, international, and well-characterized cohort of patients across all stages, using a combination of motor cardinal features (bradykinesia, rigidity, tremor, axial signs) and comprehensive NMS assessed using specific validated rater-based scales. We believe this is the largest study of its size with these characteristics, and the first to focus on exclusively NMS-based phenotyping.

## Materials and methods

### Design

Data from two independent international studies were used in the analysis: the validation study of the Non-Motor Symptoms Scale (NMSS) (*n* = 411) (Martinez-Martin et al., 2009a) and baseline data from the global Non-Motor International Longitudinal Study (NILS) (*n* = 540) (Ray Chaudhuri et al., 2013). NILS has been adopted as a national study by the National Institute of Health Research in the UK (UKCRN No: 10084) and is a 5-year follow-up study addressing the range, nature, and natural history of NMS in PD across all motor stages. All data in NMSS and NILS have been anonymized and entered into a secure database at the National Center of Epidemiology, Carlos III Institute of Health, Madrid, Spain.

### Patients

PD patients diagnosed according to internationally recognized criteria (Gibb and Lees, 1988; Lees et al., 2009) were included, and represented a mixed cohort of drug-naïve and treated PD across all disease stages. For the NMSS study, patients were older than 30 years, but for inclusion of NILS patients there was no age limit. Exclusion criteria were: inability to read, understand, or answer written questionnaires; comorbidity, sequelae, or any disorder interfering with the assessment of PD; and inability to give informed consent. Patient recruitment was carried out across 15 countries in America, Asia, and Europe from 2007 to 2011.

### Assessments

For all patients, socio-demographic and historical data were recorded and the following assessments were applied:

1. The Scales for Outcomes in Parkinson's Disease-Motor (SCOPA-Motor), a scale with three dimensions: A. Examination (10 items); B. Activities of daily living (7 items); and C. Complications (4 items). Each item scores from 0 (normal) to 3 (severe), the total score ranging from 0 to 75. This scale was derived from the Unified Parkinson's Disease Rating Scale and showed high correlation with the original scale (*r* > 0.85) and satisfactory clinimetric attributes in validation studies (Marinus et al., 2004; Martinez-Martin et al., 2005).
2. The NMSS, a 30-item scale with nine domains: cardiovascular (2 items), sleep/fatigue (4 items), mood/apathy (6 items), perceptual problems/hallucinations (3 items), attention/memory (3 items), gastrointestinal tract (3 items), urinary function (3 items), sexual function (2 items), and miscellaneous (4 items). Each item scores from 0 to 12 (severity, 0–3, multiplied by frequency, 1–4) and the total NMSS score varies from 0 to 360, a value representing the total non-motor symptomatic burden (Chaudhuri et al., 2007; Martinez-Martin et al., 2009a).
3. The Hoehn and Yahr (HY) scale (Hoehn and Yahr, 1967).
4. The Clinical Impression of Severity Index for PD (CISI-PD), a global evaluation of motor signs, disability, motor complications, and cognitive status. Items are rated from 0 (normal) to 6 (very severe), the total score ranging from 0 to 24 (Martinez-Martin et al., 2006, 2009b).

### Standard protocol approvals, registrations, and patient consent

The NMSS validation study received ethical approval from the Carlos III Institute of Health, Madrid, Spain, and local research ethics committees (Martinez-Martin et al., 2009a). The NILS is included in the UK Department of Health portfolio of approved studies (UK CRN portfolio Nr. 10084) and has been approved at all relevant institutions and corresponding ethics committees/institutional review boards. All patients gave written informed consent before inclusion in accordance with the Declaration of Helsinki.

### Statistical analysis

SCOPA-Motor examination items were aggregated to obtain four “cardinal motor signs”: tremor (items 1 and 2), bradykinesia (item 3), rigidity (item 4), and axial signs (items 5 to 10). Additionally, an aggregate “motor complications” variable was obtained from the sum of items 18 to 21 (dyskinesias and motor fluctuations). All variables were standardized before clustering, and unstandardized afterwards for interpretation. Analyses were conducted in R version 3.2.4 (www.r-project.org) and Stata version 14 (http://www.stata.com/).

#### Cluster analysis

*k*-means was used for cluster analysis. We performed two analyses on the data: the first clustering on the nine aggregate non-motor symptom domains, the four cardinal motor signs (tremor, bradykinesia, rigidity, axial), and motor complications, henceforth the “domains clustering,” and the second on the 30 individual NMS of the NMSS only, henceforth the “symptoms clustering.” Average-linkage hierarchical agglomerative clustering on the 30 NMS, 4 motor signs, and motor complications was also performed to observe the grouping of the variables.

Various formal measures were used to determine the optimal number of clusters for the dataset. For the domains clustering, the optimal *k* according to the Gap Statistic and the 1-standard-error method (Tibshirani et al., 2001) was *k* = 4 (Supplementary Figure 1A). Other cluster determination methods suggested *k* = 2, 3, 4, where *k* = 2, 3 simply divided the data uninformatively into groups with varying levels of overall disease severity. Thus, *k* = 4 was selected to offer a good combination of model fit and parsimony. The same method was applied for the symptoms clustering, where the number of clusters was *k* = 6 (Supplementary Figure 1B).

#### Comparative subgroup analysis

For each variable in both clusterings, we used one-way ANOVA and χ^2^ tests to, respectively, check the equality of variable means and proportions across the clusters found, using Bonferroni correction for multiple testing with corrected *p* < 0.05 considered significant. Differences among pairwise clusters were tested *post-hoc* using Tukey's range test for continuous means, or pairwise χ^2^ tests for proportions, with Bonferroni correction both for the within-variable pairwise tests and the multiple variable comparisons.

To compare the domains and symptoms clusterings, we depicted cluster alignment with a contingency table, and computed the adjusted rand index (ARI) (Hubert and Arabie, 1985) to evaluate similarity between the two clusterings.

Lastly, to explore the relationship between symptom severity and disease duration, we computed the correlation of each variable with disease duration and fitted smoothed loess curves to the data both globally and for each cluster in the domains clustering.

## Results

### Study sample

Out of the 951 patients in the study, we used list wise deletion to exclude 47 patients due to missing measurements, resulting in 904 remaining patients. There were no significant differences between the included and excluded groups with respect to age, sex, disease duration, and HY (χ^2^ ≥ 0.19). The characteristics of the sample included for analysis (*n* = 904) are displayed in Table 1. Patients were predominantly male (62.17%). 13.38% were in HY stage 1; 43.36% in stage 2; 29.65% in stage 3; 11.50% in stage 4; and 2.10% in stage 5.

**Table 1:** Description of the sample.

	**Mean**	***SD***	**Median**	**Range**
Age at study	64.28	9.86	65	34–89
Age at onset of Parkinson's disease (PD onset)	56.27	10.72	57	25–89
Duration of the disease (PD duration)	8.01	5.80	7	0–40
Non-Motor symptoms scale total score	50.45	41.72	39	0–225
1. Cardiovascular	1.74	3.26	0	0–24
2. Sleep/Fatigue	8.76	8.71	6	0–48
3. Mood/Apathy	8.67	11.54	4	0–60
4. Perceptual problems/Hallucinations	1.64	3.86	0	0–33
5. Attention/Memory	5.40	7.42	2	0–36
6. Gastrointestinal	5.53	6.78	3	0–36
7. Urinary	8.07	8.93	5	0–36
8. Sexual function	3.53	5.98	0	0–24
9. Miscellaneous	7.12	7.78	4	0–48
Cardinal motor features^*^				
Tremor	2.59	2.58	2	0–12
Bradykinesia	2.40	1.41	2	0–6
Rigidity	2.23	1.36	2	0–6
Axial	3.25	2.67	3	0–12
SCOPA-Motor total score	21.07	12.06	19	1–72
A. Examination	11.54	6.56	10	0–41
B. Activities of daily living	6.80	4.19	7	0–21
C. Complications	2.73	3.01	2	0–12
Clinicial Impression of Severity Index (CISI-PD)	8.29	4.61	8	0–24

*SD: Standard deviation. SCOPA: Scales for Outcomes in Parkinson's Disease*.

* *Scores derived from items of the SCOPA-Motor A. Examination*.

### Domains clustering

**Figure 1:**
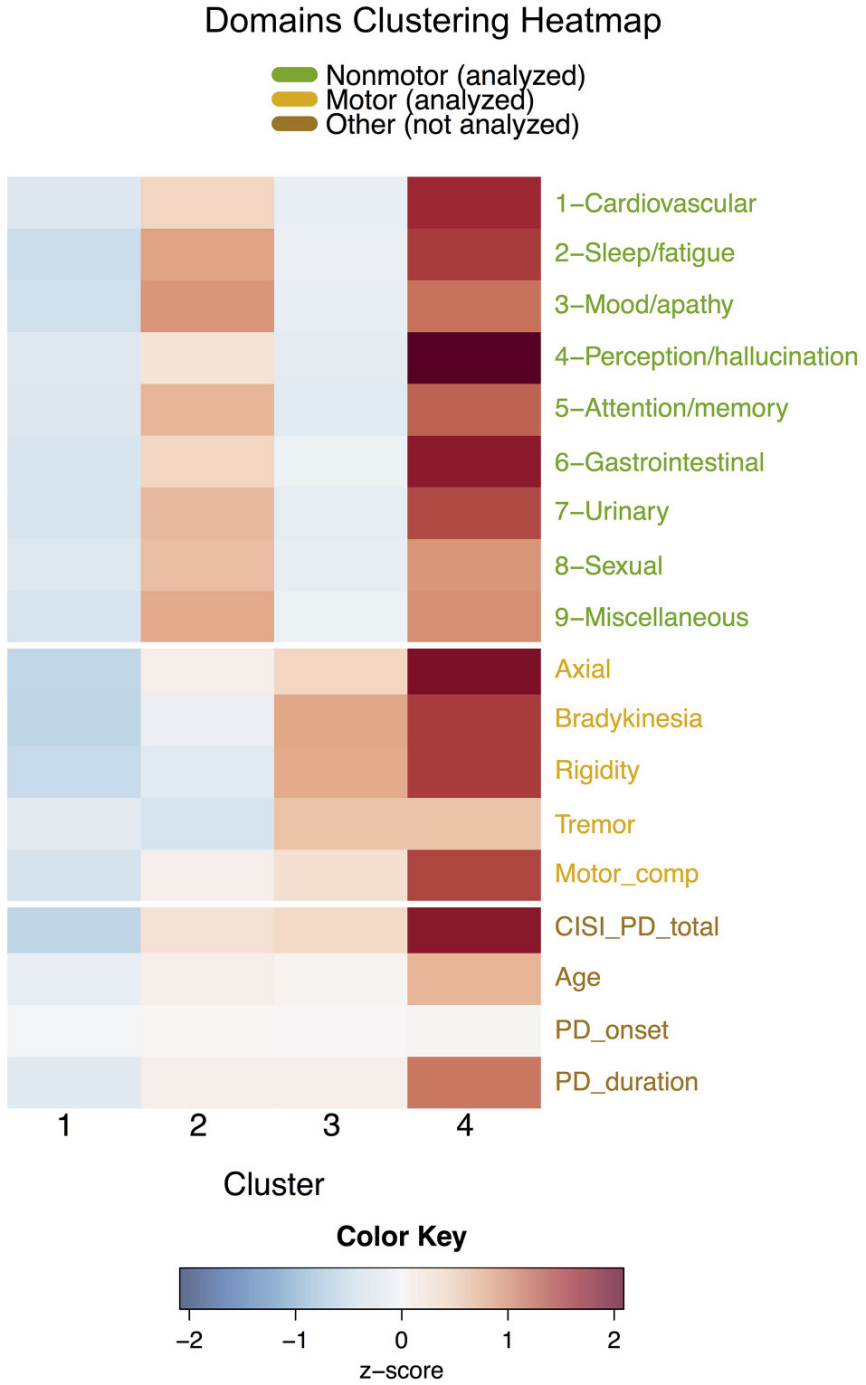
Heatmap of variables for each cluster in the domains clustering, separated by white lines according to nine non-motor domains, four cardinal motor features, motor complications, and four general variables not included in the analyses. Since symptoms have different scales, cluster means for each symptom are displayed as standardized scores relative to each overall symptom mean.

Results from the *k*-means clustering on the nine non-motor domains, the four cardinal motor signs, and motor complications are reported in Table 2 along with additional variables not used in the analysis (heatmap in Figure 1; boxplots in Supplementary Figure 2). Cluster means for all variables were found to be statistically significantly different except for age at disease onset and sex (adjusted *p* < 0.05). Specific pairwise differences are noted in the table.

**Table 2:** Domains clustering summary.

	**Cluster**	**D1**	**D2**	**D3**	**D4**
	*n* (%)	428 (47%)	180 (20%)	232 (26%)	64 (7%)
Non-motor domains	1. Cardiovascular	0.7 (1.5)^2^^,^ ^4^	3.2 (3.7)^1^^,^ ^3^^,^ ^4^	1.1 (2.1)^2^^,^ ^4^	6.9 (6.4)^1^^,^ ^2^^,^ ^3^
	2. Sleep/fatigue	4.5 (5.0)^2^^,^ ^3^^,^ ^4^	16 (8.7)^1^^,^ ^3^^,^ ^4^	7.5 (6.6)^1^^,^ ^2^^,^ ^4^	21.7 (9.7)^1^^,^ ^2^^,^ ^3^
	3. Mood/apathy	3.4 (4.8)^2^^,^ ^3^^,^ ^4^	19.2 (15.0)^1^^,^ ^3^	6.6 (8.0)^1^^,^ ^2^^,^ ^4^	21.7 (13.5)^1^^,^ ^3^
	4. Perception/hallucination	0.5 (1.7)^2^^,^ ^4^	2.7 (4.3)^1^^,^ ^3^^,^ ^4^	0.8 (1.8)^2^^,^ ^4^	9.7 (6.9)^1^^,^ ^2^^,^ ^3^
	5. Attention/memory	3.0 (4.5)^2^^,^ ^4^	10.5 (9.2)^1^^,^ ^3^^,^ ^4^	3.3 (4.4)^2^^,^ ^4^	14.5 (11.0)^1^^,^ ^2^^,^ ^3^
	6. Gastrointestinal	2.9 (4.1)^2^^,^ ^3^^,^ ^4^	8.5 (7.1)^1^^,^ ^3^^,^ ^4^	4.7 (5.3)^1^^,^ ^2^^,^ ^4^	17.4 (9.2)^1^^,^ ^2^^,^ ^3^
	7. Urinary	4.7 (6.2)^2^^,^ ^4^	14.0 (9.9)^1^^,^ ^3^^,^ ^4^	6.2 (6.7)^2^^,^ ^4^	20.3 (9.7)^1^^,^ ^2^^,^ ^3^
	8. Sexual function	1.7 (3.4)^2^^,^ ^3^^,^ ^4^	7.3 (7.8)^1^^,^ ^3^	2.4 (4.1)^1^^,^ ^2^^,^ ^4^	9.0 (9.9)^1^^,^ ^3^
	9. Miscellaneous	4.0 (4.8)^2^^,^ ^3^^,^ ^4^	13.2 (8.7)^1^^,^ ^3^	6.2 (6.8)^1^^,^ ^2^^,^ ^4^	14.5 (10.1)^1^^,^ ^3^
Motor symptoms	Axial	1.7 (1.5)^2^^,^ ^3^^,^ ^4^	3.6 (2.2)^1^^,^ ^3^^,^ ^4^	4.5 (2.3)^1^^,^ ^2^^,^ ^4^	8.2 (2.7)^1^^,^ ^2^^,^ ^3^
	Bradykinesia	1.6 (0.9)^2^^,^ ^3^^,^ ^4^	2.2 (1.1)^1^^,^ ^3^^,^ ^4^	3.5 (1.0)^1^^,^ ^2^^,^ ^4^	4.5 (1.3)^1^^,^ ^2^^,^ ^3^
	Rigidity	1.5 (0.9)^2^^,^ ^3^^,^ ^4^	1.8 (1.2)^1^^,^ ^3^^,^ ^4^	3.3 (1.0)^1^^,^ ^2^^,^ ^4^	4.2 (1.2)^1^^,^ ^2^^,^ ^3^
	Tremor	2.0 (1.9)^3^^,^ ^4^	1.5 (1.8)^3^^,^ ^4^	4.1 (2.8)^1^^,^ ^2^	4.1 (4.1)^1^^,^ ^2^
	Motor complications	1.4 (2.1)^2^^,^ ^3^^,^ ^4^	3.1 (2.9)^1^^,^ ^4^	3.7 (2.9)^1^^,^ ^4^	7.0 (3.6)^1^^,^ ^2^^,^ ^3^
Variables not used in analysis	Sex (% male)	64	54	67	58
	CISI-PD total	5.5 (3.0)^2^^,^ ^3^^,^ ^4^	9.6 (3.8)^1^^,^ ^4^	10.1 (3.5)^1^^,^ ^4^	16.4 (4.6)^1^^,^ ^2^^,^ ^3^
	Age	62.5 (9.7)^4^	65.2 (9.4)^4^	64.9 (10.1)^4^	71.1 (7.9)^1^^,^ ^2^^,^ ^3^
	PD onset	56 (10.5)	56.6 (10.6)	56.3 (11.3)	56.7 (10.6)
	PD duration	6.5 (4.7)^2^^,^ ^3^^,^ ^4^	8.6 (5.7)^1^^,^ ^4^	8.6 (5.7)^1^^,^ ^4^	14.4 (8.0)^1^^,^ ^2^^,^ ^3^

*Unless otherwise specified, statistics are reported as mean (sd)*.

1 *Significant difference with cluster D1 (corrected p < 0.05)*.

2 *Significant difference with cluster D2 (corrected p < 0.05)*.

3 *Significant difference with cluster D3 (corrected p < 0.05)*.

4 *Significant difference with cluster D4 (corrected p < 0.05)*.

Cluster D1 (*n* = 428) patients were mildly affected in all domains. This cluster was characterized by relatively lower disease durations and ages.

Cluster D2 (*n* = 180) patients were severely affected in non-motor domains but mildly affected in motor domains. This cluster had a severity of motor variables relatively similar to the cluster D1 (mild) subtype especially in tremor, but expressed significantly higher scores for non-motor domains than clusters D1 and D3, especially in the sleep/fatigue, mood/apathy, urinary, and miscellaneous domains. Except for motor complications, scores for every variable were statistically significantly different from those in cluster D3.

Cluster D3 (*n* = 232) patients were severely affected in motor domains but mildly affected in non-motor domains. Mean motor scores were greater than the means of clusters D1 and D2, with the exception of motor complications. Additionally, mean motor scores were less than D4, with the exception of tremor, which was especially high. Importantly, CISI-PD scores of clusters D2 and D3 were not statistically significantly different, and no differences were observed in cluster D2 and cluster D3 age or disease duration.

Cluster D4 (*n* = 64) patients were severely affected in all domains, having the greatest symptom mean out of all four clusters with the exception of tremor. Consequently, patients in cluster D4 had the longest average disease duration and oldest ages, but did not have a significantly different age of disease onset.

### Symptoms clustering

**Figure 2:**
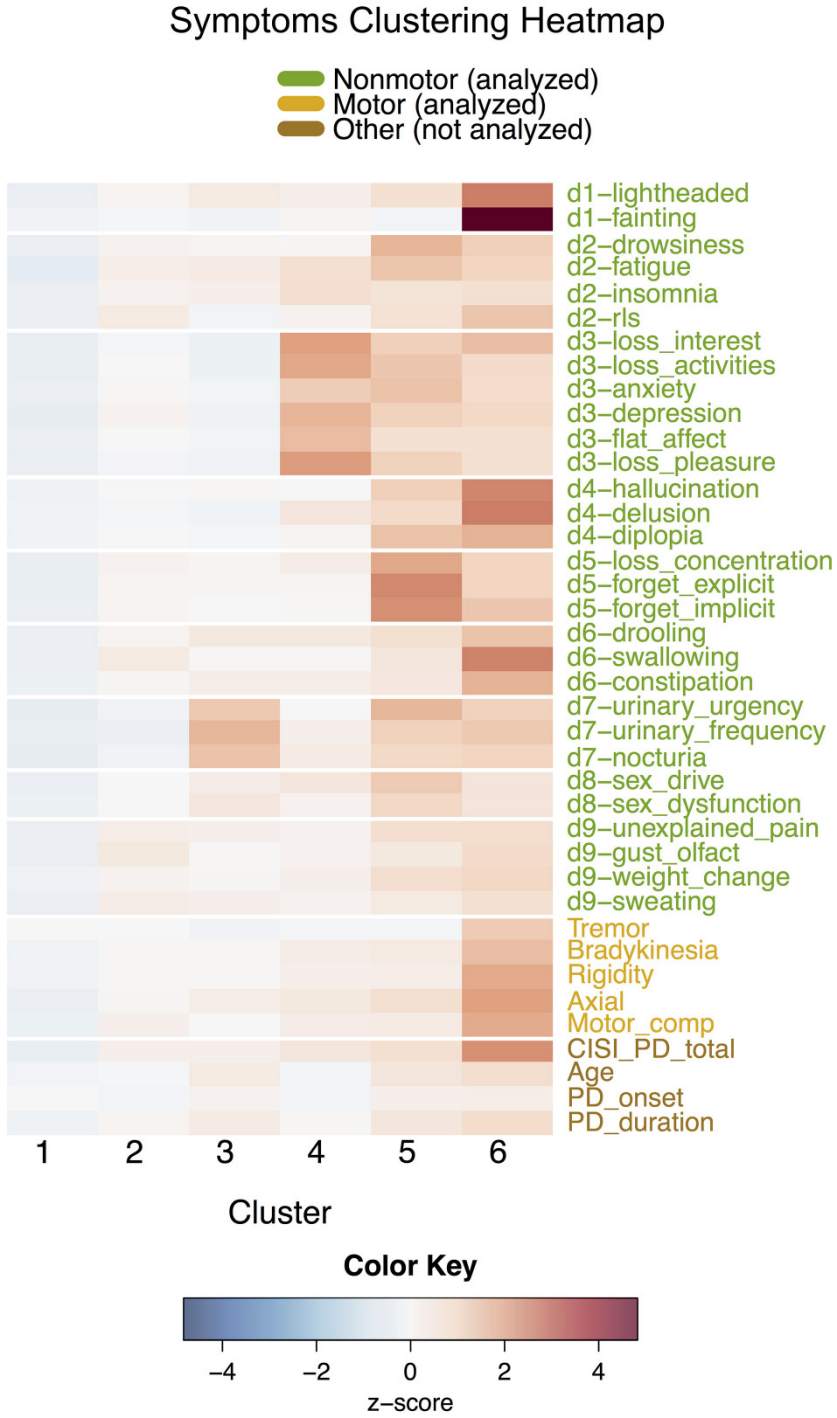
Heatmap of variables for each cluster in the symptoms clustering, separated by white lines according to 30 individual non-motor symptoms and variables not included in the analysis (cardinal motor features, motor complications, and other general variables). Since symptoms have different scales, cluster means for each symptom are displayed as standardized scores relative to each overall symptom mean.

*k*-means performed on the 30 individual NMS found six clusters ordered according to increasing CISI-PD score (Table 3, heatmap in Figure 2). Means of all symptoms were found to differ across clusters except for disease onset, sex, and tremor, with pairwise differences again noted in the table.

**Table 3:** Symptoms clustering summary.

	**Cluster**	**S1**	**S2**	**S3**	**S4**	**S5**	**S6**
	*n* (%)	456 (50%)	201 (22%)	100 (11%)	73 (8%)	54 (6%)	20 (2%)
1. Cardiovascular	Lightheadedness	0.5 (1.1)^2^^,^ ^3^^,^ ^4^^,^ ^5^^,^ ^6^	1.7 (2.5)^1^^,^ ^5^^,^ ^6^	2.5 (3.4)^1^^,^ ^6^	1.9 (2.5)^1^^,^ ^6^	3.3 (3.7)^1^^,^ ^2^^,^ ^6^	7.7 (3.6)^1^^,^ ^2^^,^ ^3^^,^ ^4^^,^ ^5^
	Fainting	0.1 (0.6)^6^	0.3 (1)^6^	0.1 (0.5)^6^	0.5 (1.2)^6^	0.1 (0.5)^6^	6.3 (2.9)^1^^,^ ^2^^,^ ^3^^,^ ^4^^,^ ^5^
2. Sleep/fatigue	Drowsiness	0.9 (1.8)^2^^,^ ^3^^,^ ^4^^,^ ^5^^,^ ^6^	2.4 (2.9)^1^^,^ ^5^^,^ ^6^	2.3 (2.9)^1^^,^ ^5^^,^ ^6^	2.2 (3)^1^^,^ ^5^^,^ ^6^	6.6 (4.2)^1^^,^ ^2^^,^ ^3^^,^ ^4^	5.2 (3)^1^^,^ ^2^^,^ ^3^^,^ ^4^
	Fatigue	1.2 (1.9)^2^^,^ ^3^^,^ ^4^^,^ ^5^^,^ ^6^	4.2 (3.7)^1^^,^ ^4^^,^ ^5^	4.5 (4)^1^^,^ ^5^	5.9 (4.2)^1^^,^ ^2^	8 (3.5)^1^^,^ ^2^^,^ ^3^	6.9 (3.1)^1^
	Insomnia	1.1 (2.2)^2^^,^ ^3^^,^ ^4^^,^ ^5^^,^ ^6^	3 (3.8)^1^^,^ ^4^	3.1 (4.2)^1^^,^ ^4^	5.3 (4.8)^1^^,^ ^2^^,^ ^3^	4.7 (4.8)^1^	5 (2.4)^1^
	RLS	0.5 (1.3)^2^^,^ ^4^^,^ ^5^^,^ ^6^	2.4 (3.5)^1^^,^ ^3^^,^ ^6^	1 (2.4)^2^^,^ ^5^^,^ ^6^	1.8 (3.2)^1^^,^ ^6^	3.2 (4.2)^1^^,^ ^3^	4.9 (2.6)^1^^,^ ^2^^,^ ^3^^,^ ^4^
3. Mood/apathy	Loss interest	0.4 (0.9)^2^^,^ ^4^^,^ ^5^^,^ ^6^	1.1 (1.8)^1^^,^ ^4^^,^ ^5^^,^ ^6^	0.6 (1.3)^4^^,^ ^5^^,^ ^6^	6.6 (3.9)^1^^,^ ^2^^,^ ^3^^,^ ^5^	4.5 (3.7)^1^^,^ ^2^^,^ ^3^^,^ ^4^	5.3 (2.8)^1^^,^ ^2^^,^ ^3^
	Loss activities	0.6 (1.3)^2^^,^ ^4^^,^ ^5^^,^ ^6^	1.9 (2.7)^1^^,^ ^4^^,^ ^5^^,^ ^6^	1 (2)^4^^,^ ^5^^,^ ^6^	7.8 (3.5)^1^^,^ ^2^^,^ ^3^^,^ ^5^^,^ ^6^	6 (4.5)^1^^,^ ^2^^,^ ^3^^,^ ^4^	4.7 (2.9)^1^^,^ ^2^^,^ ^3^^,^ ^4^
	Anxiety	0.8 (1.6)^2^^,^ ^4^^,^ ^5^^,^ ^6^	2.2 (2.9)^1^^,^ ^4^^,^ ^5^^,^ ^6^	1.6 (2.9)^4^^,^ ^5^^,^ ^6^	5.8 (4.3)^1^^,^ ^2^^,^ ^3^	6.4 (4.4)^1^^,^ ^2^^,^ ^3^	4.7 (3)^1^^,^ ^2^^,^ ^3^
	Depression	0.7 (1.4)^2^^,^ ^4^^,^ ^5^^,^ ^6^	2.7 (3.2)^1^^,^ ^3^^,^ ^4^^,^ ^5^^,^ ^6^	1.4 (2.3)^2^^,^ ^4^^,^ ^5^^,^ ^6^	7.4 (4)^1^^,^ ^2^^,^ ^3^	5.8 (4.3)^1^^,^ ^2^^,^ ^3^	5.3 (3.2)^1^^,^ ^2^^,^ ^3^
	Flat affect	0.3 (1)^2^^,^ ^4^^,^ ^5^^,^ ^6^	1.1 (2.1)^1^^,^ ^4^^,^ ^5^	0.8 (2.1)^4^^,^ ^5^^,^ ^6^	4.8 (4.3)^1^^,^ ^2^^,^ ^3^^,^ ^5^	2.9 (3.7)^1^^,^ ^2^^,^ ^3^^,^ ^4^	3 (2.1)^1^^,^ ^3^
	Loss pleasure	0.3 (1.1)^4^^,^ ^5^^,^ ^6^	1 (1.8)^4^^,^ ^5^^,^ ^6^	0.8 (1.8)^4^^,^ ^5^^,^ ^6^	7.3 (3.8)^1^^,^ ^2^^,^ ^3^^,^ ^5^^,^ ^6^	4.6 (4.2)^1^^,^ ^2^^,^ ^3^^,^ ^4^	3.6 (2.8)^1^^,^ ^2^^,^ ^3^^,^ ^4^
4. Perception/hallucination	Hallucination	0.2 (0.9)^5^^,^ ^6^	0.6 (1.7)^5^^,^ ^6^	0.7 (1.9)^5^^,^ ^6^	0.6 (1.8)^5^^,^ ^6^	2.7 (3.3)^1^^,^ ^2^^,^ ^3^^,^ ^4^^,^ ^6^	4.8 (3.3)^1^^,^ ^2^^,^ ^3^^,^ ^4^^,^ ^5^
	Delusion	0.1 (0.6)^4^^,^ ^5^^,^ ^6^	0.3 (1.4)^4^^,^ ^5^^,^ ^6^	0.1 (0.6)^4^^,^ ^5^^,^ ^6^	1.3 (3)^1^^,^ ^2^^,^ ^3^^,^ ^6^	2 (3.5)^1^^,^ ^2^^,^ ^3^^,^ ^6^	4.7 (3.4)^1^^,^ ^2^^,^ ^3^^,^ ^4^^,^ ^5^
	Diplopia	0.2 (0.8)^5^^,^ ^6^	0.6 (1.4)^5^^,^ ^6^	0.4 (1.6)^5^^,^ ^6^	0.8 (2.2)^5^^,^ ^6^	3.1 (4.4)^1^^,^ ^2^^,^ ^3^^,^ ^4^	3.6 (2.7)^1^^,^ ^2^^,^ ^3^^,^ ^4^
5. Attention/memory	Loss concentration	0.7 (1.5)^2^^,^ ^3^^,^ ^4^^,^ ^5^^,^ ^6^	2.6 (2.9)^1^^,^ ^5^^,^ ^6^	2.2 (3.4)^1^^,^ ^5^^,^ ^6^	2.9 (2.9)^1^^,^ ^5^	7.5 (3.9)^1^^,^ ^2^^,^ ^3^^,^ ^4^	5.1 (2.8)^1^^,^ ^2^^,^ ^3^
	Forget explicit	0.7 (1.3)^2^^,^ ^3^^,^ ^4^^,^ ^5^^,^ ^6^	2.2 (2.7)^1^^,^ ^5^^,^ ^6^	2.2 (3)^1^^,^ ^5^^,^ ^6^	2.1 (2.5)^1^^,^ ^5^^,^ ^6^	8.5 (3.2)^1^^,^ ^2^^,^ ^3^^,^ ^4^^,^ ^6^	4.8 (2.9)^1^^,^ ^2^^,^ ^3^^,^ ^4^^,^ ^5^
	Forget implicit	0.5 (1.2)^2^^,^ ^3^^,^ ^4^^,^ ^5^^,^ ^6^	1.9 (2.6)^1^^,^ ^5^^,^ ^6^	1.5 (2.7)^1^^,^ ^5^^,^ ^6^	1.8 (2.2)^1^^,^ ^5^^,^ ^6^	7.6 (4.1)^1^^,^ ^2^^,^ ^3^^,^ ^4^^,^ ^6^	5.1 (3.2)^1^^,^ ^2^^,^ ^3^^,^ ^4^^,^ ^5^
6. Gastrointestinal	Drooling	0.6 (1.5)^2^^,^ ^3^^,^ ^4^^,^ ^5^^,^ ^6^	2.3 (3.3)^1^^,^ ^5^^,^ ^6^	3.3 (4.1)^1^^,^ ^6^	3.3 (4.2)^1^^,^ ^6^	4.2 (4.8)^1^^,^ ^2^	6.2 (3.3)^1^^,^ ^2^^,^ ^3^^,^ ^4^
	Swallowing	0.3 (0.8)^2^^,^ ^3^^,^ ^5^^,^ ^6^	2 (3)^1^^,^ ^6^	1.2 (2)^1^^,^ ^6^	1.2 (2)^6^	2.3 (2.9)^1^^,^ ^6^	6.5 (4.2)^1^^,^ ^2^^,^ ^3^^,^ ^4^^,^ ^5^
	Constipation	1.5 (2.7)^2^^,^ ^3^^,^ ^4^^,^ ^5^^,^ ^6^	3 (3.8)^1^^,^ ^6^	3.8 (4.4)^1^^,^ ^6^	3.7 (4.4)^1^^,^ ^6^	4.5 (4.9)^1^^,^ ^6^	8.8 (4.4)^1^^,^ ^2^^,^ ^3^^,^ ^4^^,^ ^5^
7. Urinary	Urinary urgency	0.9 (1.7)^2^^,^ ^3^^,^ ^4^^,^ ^5^^,^ ^6^	1.8 (2.4)^1^^,^ ^3^^,^ ^5^^,^ ^6^	6.6 (3.8)^1^^,^ ^2^^,^ ^4^	2.4 (3.4)^1^^,^ ^3^^,^ ^5^^,^ ^6^	7.7 (4.3)^1^^,^ ^2^^,^ ^4^	6.2 (3.5)^1^^,^ ^2^^,^ ^4^
	Urinary frequency	0.9 (1.8)^3^^,^ ^4^^,^ ^5^^,^ ^6^	1.3 (1.9)^3^^,^ ^4^^,^ ^5^^,^ ^6^	7.7 (3.5)^1^^,^ ^2^^,^ ^4^	3.1 (3.6)^1^^,^ ^2^^,^ ^3^^,^ ^5^^,^ ^6^	6.2 (4.5)^1^^,^ ^2^^,^ ^4^	6.7 (3.1)^1^^,^ ^2^^,^ ^4^
	Nocturia	1.7 (2.3)^3^^,^ ^4^^,^ ^5^^,^ ^6^	2.6 (2.9)^3^^,^ ^4^^,^ ^5^^,^ ^6^	8.5 (3.5)^1^^,^ ^2^^,^ ^4^	4.6 (4.2)^1^^,^ ^2^^,^ ^3^^,^ ^5^	6.9 (4.4)^1^^,^ ^2^^,^ ^4^	7.1 (3.4)^1^^,^ ^2^
8. Sexual	Sex drive	0.7 (1.7)^2^^,^ ^3^^,^ ^4^^,^ ^5^^,^ ^6^	1.9 (3.4)^1^^,^ ^5^	3 (4.1)^1^^,^ ^5^	3.6 (4.4)^1^^,^ ^5^	6 (5.3)^1^^,^ ^2^^,^ ^3^^,^ ^4^	3.9 (5.3)^1^
	Sex dysfunction	0.7 (2)^3^^,^ ^4^^,^ ^5^^,^ ^6^	1.8 (3.3)^3^^,^ ^5^	3.4 (4.3)^1^^,^ ^2^	2.3 (4.2)^1^^,^ ^5^	5 (5.2)^1^^,^ ^2^^,^ ^4^	3.8 (5.6)^1^
9. Miscellaneous	Unexplained pain	0.7 (1.8)^2^^,^ ^3^^,^ ^4^^,^ ^5^^,^ ^6^	2.6 (3.9)^1^	2.4 (4)^1^	2.3 (3.6)^1^	4.3 (5)^1^	4.3 (2.2)^1^
	Gustation/olfaction	1.2 (2.5)^2^^,^ ^4^^,^ ^5^^,^ ^6^	4 (4.2)^1^	2.5 (3.6)	3 (3.9)^1^	4 (4.7)^1^	5.5 (4.7)^1^
	Weight change	0.8 (1.4)^2^^,^ ^4^^,^ ^5^^,^ ^6^	1.9 (3.1)^1^^,^ ^5^	1.8 (3)^5^	2.1 (2.8)^1^	3.6 (4.3)^1^^,^ ^2^^,^ ^3^	4 (3.9)^1^
	Sweating	0.6 (1.6)^2^^,^ ^3^^,^ ^4^^,^ ^5^^,^ ^6^	2.5 (3.9)^1^	2.4 (3.9)^1^	2.1 (3.6)^1^	2.8 (3.9)^1^	3.9 (3.4)^1^
Motor symptoms	Axial	2.3 (2)^2^^,^ ^3^^,^ ^4^^,^ ^5^^,^ ^6^	3.5 (2.6)^1^^,^ ^5^^,^ ^6^	4 (2.8)^1^^,^ ^6^	4.4 (2.7)^1^^,^ ^6^	5.3 (3.3)^1^^,^ ^2^^,^ ^6^	8.5 (2.3)^1^^,^ ^2^^,^ ^3^^,^ ^4^^,^ ^5^
	Bradykinesia	2.1 (1.2)^4^^,^ ^5^^,^ ^6^	2.5 (1.4)^6^	2.5 (1.4)^6^	2.9 (1.4)^1^^,^ ^6^	3 (1.6)^1^^,^ ^6^	4.5 (1.7)^1^^,^ ^2^^,^ ^3^^,^ ^4^^,^ ^5^
	Rigidity	2 (1.2)^6^	2.3 (1.5)^6^	2.3 (1.4)^6^	2.5 (1.3)^6^	2.6 (1.5)^6^	4.7 (1)^1^^,^ ^2^^,^ ^3^^,^ ^4^^,^ ^5^
	Tremor	2.6 (2.4)^6^	2.5 (2.4)^6^	2.1 (2.5)^6^	2.5 (2.6)^6^	2.4 (2.8)^6^	5.8 (5.3)^1^^,^ ^2^^,^ ^3^^,^ ^4^^,^ ^5^
	Motor comp	1.9 (2.5)^2^^,^ ^4^^,^ ^5^^,^ ^6^	3.5 (3)^1^^,^ ^6^	2.7 (2.7)^6^	3.7 (3.3)^1^^,^ ^6^	3.9 (3.7)^1^^,^ ^6^	8 (3.7)^1^^,^ ^2^^,^ ^3^^,^ ^4^^,^ ^5^
Other variables not	Sex	67	57	64	49	63	50
used in analysis	CISI PD total	6.4 (3.5)^2^^,^ ^3^^,^ ^4^^,^ ^5^^,^ ^6^	9.3 (4.1)^1^^,^ ^5^^,^ ^6^	9.4 (4.1)^1^^,^ ^6^	10.7 (4.6)^1^^,^ ^6^	11.8 (5.5)^1^^,^ ^2^^,^ ^6^	18.4 (4.2)^1^^,^ ^2^^,^ ^3^^,^ ^4^^,^ ^5^
	Age	62.8 (10)^3^^,^ ^5^^,^ ^6^	63.8 (10)^5^	68.3 (7.5)^1^	63.1 (9.2)	69.7 (9.2)^1^^,^ ^2^	72.1 (7.7)^1^
	PD onset	56.2 (10.8)	55 (10.7)	58.2 (10)	54.8 (10.3)	58.8 (11.7)	59.2 (8.6)
	PD duration	6.6 (4.8)^2^^,^ ^3^^,^ ^5^^,^ ^6^	8.8 (5.9)^1^	10.1 (6.7)^1^	8.3 (4.9)	11 (8.5)^1^	12.8 (6.5)^1^

*Unless otherwise specified, statistics are reported as mean (sd)*.

1 *significant difference with cluster S1 (corrected p < 0.05)*.

2 *significant difference with cluster S2 (corrected p < 0.05)*.

3 *significant difference with cluster S3 (corrected p < 0.05)*.

4 *significant difference with cluster S4 (corrected p < 0.05)*.

5 *significant difference with cluster S5 (corrected p < 0.05)*.

6 *significant difference with cluster S6 (corrected p < 0.05)*.

Cluster S1 (*n* = 456), the largest cluster representing 50% of the group, was similar to domains cluster D1, and was composed of patients relatively mildly affected in all NMS. Cluster S2 (*n* = 201) had higher mean symptom scores than cluster S1's in several cases, including restless legs syndrome (RLS), swallowing, and the miscellaneous domain, but could nonetheless be classified as a mild/moderate cluster.

Although clusters S3–S6 increased in motor and overall disease severity, they varied significantly in their non-motor expression and each expressed a unique subset of NMS. These groups of NMS aligned well with the established non-motor domains. Cluster S3 (*n* = 100) mainly expressed domain 7 (urinary), while cluster S4 (*n* = 73) was affected severely in domain 3 (mood/apathy). Cluster S5 (*n* = 54) showed severe impact in most NMS but especially in domain 5 (attention/memory). Similarly, cluster S6 (*n* = 20) had severe scores across all NMS and motor features, but was most severely affected in the cardiovascular, perception/hallucination, and gastrointestinal NMSS domains. Overall, the symptoms clustering fragmented the domains clusters into smaller groups, as explored in the next section.

### Comparison between clusterings

Alignment of the D and S clusters is visualized in Table 4. While S1 grouped patients from D1 (mild) and D3 (motor-dominant), and D4 (severe) showed a dominant contribution from S5 (severe non-motor) and S6 (severe motor and non-motor), the remaining clusters were fragmentarily distributed, as indicated by the low similarity between the clusterings (ARI = 0.32). For the domains clustering, patients in clusters mildly affected in non-motor domains (D1, D3) were distributed among the milder symptoms clusters (S1–S4, skewed left). Conversely, patients in clusters with severe NMS (D2, D4) were split among the various specific non-motor-dominant clusters (S2–S6), suggesting that the symptoms clustering is clinically more specific than the domains clustering.

**Table 4:** Contingency table describing cross-categorization of individuals in the domains and symptoms clusters.

	**Symptoms clusters**							
**Domains clusters**		S1	S2	S3	S4	S5	S6	Total
	D1	335	64	26	3	0	0	428
	D2	0	54	46	49	31	0	180
	D3	121	77	22	12	0	0	232
	D4	0	6	6	9	23	20	64
Total		456	201	100	73	54	20	904

### Hierarchical clustering on variables

**Figure 3:**
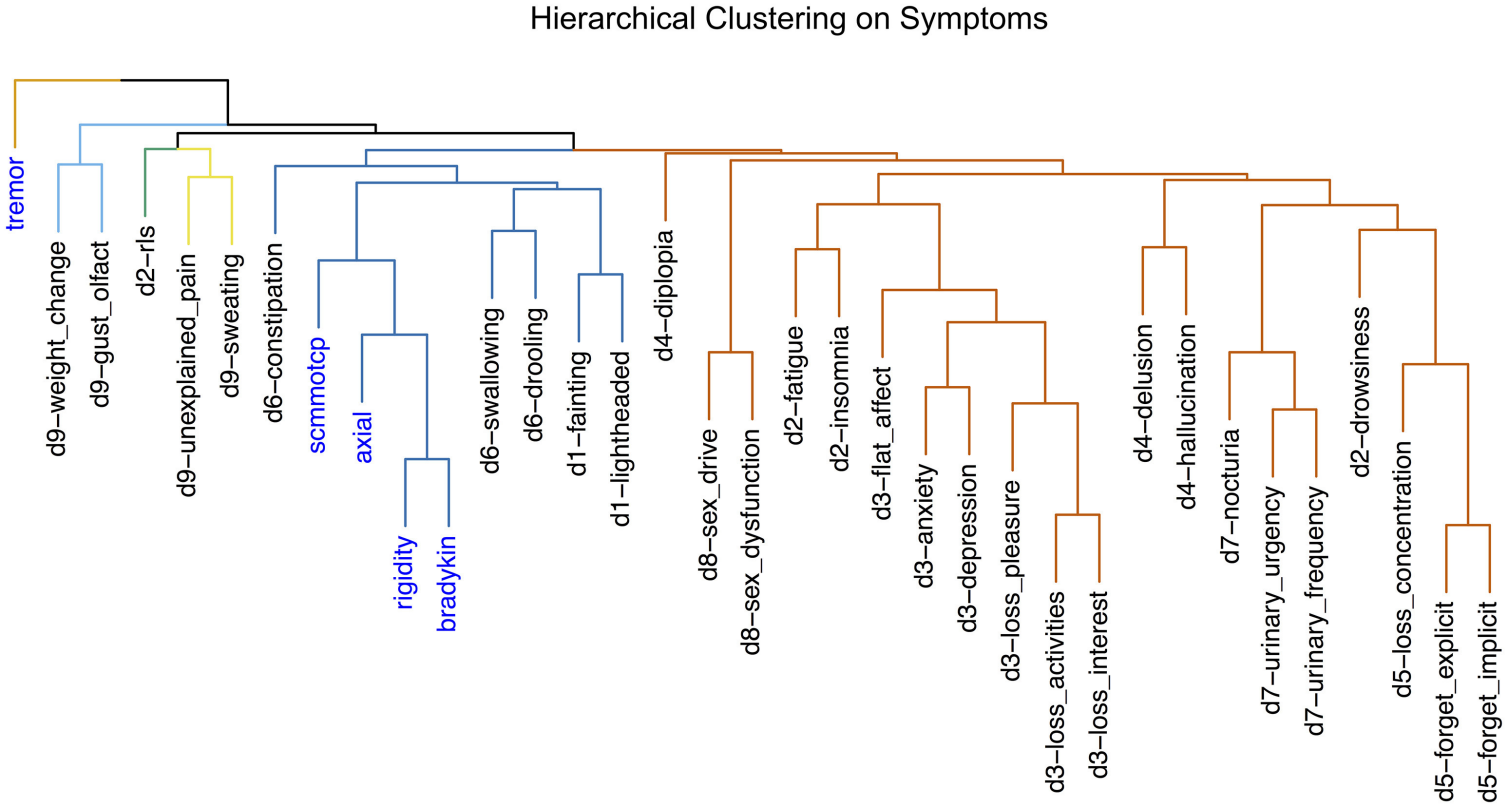
Average-linkage hierarchical clustering of motor (blue) and non-motor (black) symptoms. Symptoms are labeled with their name and corresponding domain number. The tree is colored with five clusters.

Hierarchical clustering on the 30 NMS and the four cardinal motor signs is depicted in Figure 3. Symptoms belonging to the same domain of the NMSS tended to cluster together, with some exceptions. Diplopia (domain 4) was grouped closer to domain 8 (sexual) symptoms than to symptoms in its own domain. Similarly, RLS (domain 2) was closer to domain 9 (miscellaneous) symptoms, and drowsiness (domain 2) with domain 5 (attention/memory) symptoms. Notably, tremor was the most isolated symptom, occupying a single branch at the top of the tree.

### Correlation analysis

**Figure 4:**
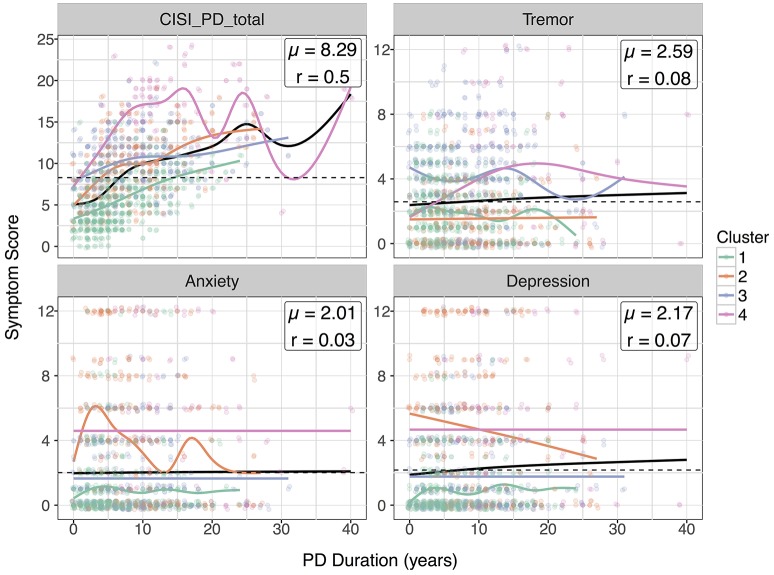
Scatterplots of selected symptoms against disease duration. For clarity, scatterplot points are colored according to cluster and jittered slightly. Smoothed loess curves for each cluster are drawn in their respective cluster colors. The black curve is the curve for the entire population, and the global mean score is marked with a dotted line.

Due to high variance, most variables had little to no correlation with disease duration (Supplementary Figure 3). In Figure 4, we plotted 4 variables especially relevant to the domains clustering against disease progression: CISI-PD, Tremor, Anxiety, and Depression. Notable differences in disease progression for each cluster can be seen in the scatterplots: for example, patients in NMS dominant cluster D2 actually tended to have higher scores for anxiety and depression at disease onset, decreasing with increasing disease duration.

## Discussion

We believe that this is the largest cluster analysis-based study of PD-related motor and NMS from a large, international, multi-center cohort. Previous cluster-analysis based studies have either focused on early/untreated Parkinson's disease (Erro et al., 2013; Pont-Sunyer et al., 2015) or lack detailed assessments based on the severity and frequency of non-motor domains and symptoms (van Rooden et al., 2011). Additionally, we believe this is the first study to perform cluster analysis exclusively on NMS to reveal NMS-specific subtypes.

The domains clustering's four clusters closely correspond with several previous studies (van Rooden et al., 2011; Erro et al., 2013; Ma et al., 2015; Pont-Sunyer et al., 2015), especially those reported by van Rooden et al. (2011). Both clusters D1 (mild) and D4 (severe) are groups which are present in most analyses, but unlike van Rooden et al., our data show that mean differences in disease duration *do* exist between mild and severe subtypes. Clusters D2 and D3 represent a divergence in symptomatic expression: D2 representing a non-motor dominant phenotype also described in many clinical phenotype-driven studies (Sauerbier et al., 2016), and D3 corresponding to the traditional motor-dominant view of PD. Due to these clusters' similar overall PD severity (CISI-PD) and duration, differences in disease progression do not explain the differences between D2 and D3. Finally, the high incidence of tremor in D3, even higher than D4, is interesting and reflects not only the motor-dominant subtype of van Rooden et al. but also the tremor-dominant/slow-progression cluster described by Ma et al. (2015).

Our correlation analysis demonstrates notable differences in disease progression among these clusters. The high initial depression and anxiety scores for cluster D2 suggests that patients susceptible to NMS-dominant PD can be identified by high NMS scores early after disease onset. Furthermore, the general improvement in depression and anxiety scores for this cluster contrasts with the relatively stable scores in clusters D1, D3, and D4.

From the symptoms clustering (Figure 2), six smaller clusters were identified. S1 was similar to D1. S2 to S6, while increasing in motor severity, expressed specific NMS, thus supporting the clinical concept of NMS-based subtyping. Cluster S2, with principal components including RLS, swallowing, pain, and others, may be a new finding from this study. Cluster S3, with significant urinary dysfunction, fits the descriptions by Erro et al. (2013), highlighting the relevance of this symptom as a specific marker in non-motor dominant clusters and disease progression. Cluster S4, characterized by high mood/apathy symptoms, is consistent with the sleep and apathy clinical phenotypes described by other studies (Sauerbier et al., 2016). Clusters S5 and S6 are of clinical interest, as in these clusters NMS dominate, overshadowing motor symptoms with an emphasis on cognitive impairment in S5 and autonomic (cardiovascular and gastrointestinal) symptoms in S6. Overall, many of these subtypes are newly reported and their characteristics support clinical endophenotyping of non-motor subtypes not reported in previous studies.

The comparison between the domains and symptoms clustering shown in the contingency table (Table 4) suggests that the broader subset of cluster S1, a mild non-motor dominant cluster, essentially expresses two NMS subtypes, one of them with motor symptoms. The low numbers observed in some cells do not allow consistent clinical interpretation. The hierarchical clustering (Figure 3) indicates that the symptoms grouping in the NMSS dimensions works as expected, as most items in each domain group together, with the exception of tremor.

What are the clinical implications of these clusterings? First, our analysis represents statistical conformation of NMS-dominant presentation of PD. The specific expression of several NMS domains such as, mood/anxiety, sleep/fatigue, cognition, and urinary function suggests that these subgroups may have different patterns of neurodegeneration involving the brain's various non-dopaminergic pathways, possibly in excess of dopaminergic degeneration, as suggested by several authors (Jellinger, 2012). Second, clinical recognition of subtypes using ad hoc criteria would allow for the development of truly subtype-specific treatment packages for PD (Marras and Chaudhuri, 2016). Third, clinical characterization of these groups will allow studies of natural history of specific subtypes.

The clinical non-specificity of D1, S1, D4, and S6, with extremely diverse disease durations and severities, contrasts with the precisely characterized clusters S3, S4, and S5, with dominant expression of urinary, mood/apathy and attention/memory symptoms, respectively, at intermediate stages of the disease. This pattern is in line with the notion of “phenotypic convergence” proposed by Warren et al. (2013) as a key clinical feature of the spread of neurodegenerative disorders due to abnormal protein aggregates. The identified clusters may represent distinct footprints of large-scale network disintegration which necessitates translation to clinical management. The Warren et al. concept is in line with the general etiologic hypothesis for late-life neurodegenerative diseases proposed by de Pedro-Cuesta et al. (2016).

Like any cohort-based, cluster-analysis driven study, there are several limitations of this analysis. Due to the data collection methods of the two studies used, selection due to prevalence bias, i.e., sample overrepresentation of patients with higher survival, is unlikely to explain this clustering; however, clustering at early PD stages may have been undermined by poorly recorded symptoms prior to diagnosis. Furthermore, we did not report a control group, although our intention was not to describe the symptoms as discriminant from normal subjects. Lastly, in the treated patients in our sample, NMS symptoms could be influenced by dopaminergic therapy, including depression via pramipexole (Barone et al., 2010), sleep disorders via rotigotine (Trenkwalder et al., 2011), and others. The numbers of patients undergoing specific treatments are too small to conduct meaningful analyses of the effects of such therapies; we expect, however, that such effects on single non-motor components do not significantly alter the trends observed in total NMSS scores across all treated and drug-naïve patients in our sample.

Conversely, our study has several notable strengths: (1) the sample size, which to our knowledge is the largest international sample in this kind of study; (2) the inclusion of patients in all disease stages; and (3) the use of detailed assessments both for motor and NMS.

In conclusion, we present statistical confirmation of the growing recognition of NMS-dominant presentation of PD and its heterogeneity. The clinical recognition of these subtypes could allow for subtype-specific treatment packages for PD, and in the future, clinical characterization of these groups will allow for studies of natural history of the various non-motor dominant clusters identified in this paper. Translating results to clinical management or experimental designs would require the identification of inclusion and exclusion criteria of patients into specific subgroups.

## Author contributions

JM conducted the statistical analysis and interpretation of the data, drafted the methods and results section of the paper, and revised the manuscript for content. KC obtained the data, designed the study, and drafted and revised the introduction and conclusion sections of the paper. CB, JdP, and PL revised the manuscript for content and contributed to the analysis and interpretation of data. PM obtained the data, designed the study, and drafted and revised the introduction and conclusion sections of the paper.

### Conflict of interest statement

The authors declare that the research was conducted in the absence of any commercial or financial relationships that could be construed as a potential conflict of interest.
