# Supplementary material for: Parkinson's Disease Subtypes Identified from Cluster Analysis of Motor and Non-motor Symptoms
Source: Front Aging Neurosci. 2017 Sep 20;9:301. doi: 10.3389/fnagi.2017.00301 (PMC5611404; doi:10.3389/fnagi.2017.00301)
Supplement: Supplementary file 1 [file Presentation1.PDF]

Supplementary Material

Parkinson's Disease Subtypes Identified from Cluster Analysis of Motor and Non-motor Symptoms

Jesse Mu, Kallol Ray Chaudhuri*, Concha Bielza, Jesus de Pedro-Cuesta, Pedro Larrañaga, Pablo Martinez-Martin

*** Correspondence:** Dr. Kallol Ray Chaudhuri: ray.chaudhuri@nhs.net

1 Supplementary Figures

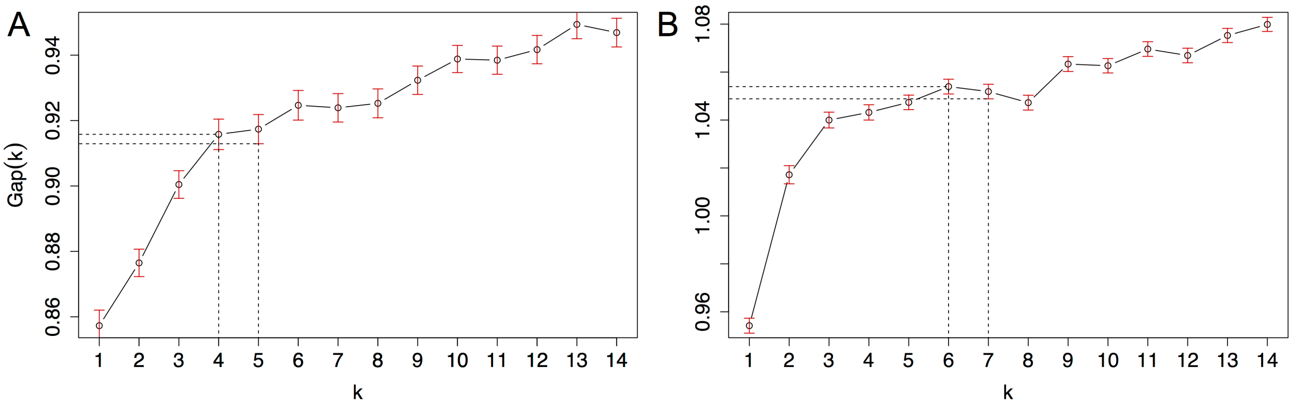

Supplementary Figure 1. Plot of the gap statistic $\mathrm{Gap}(k)$ versus number of clusters with k-means on 500 bootstrapped samples of a) the domains clustering, and b) the symptoms clustering. Error bars represent ± 1 standard error (se). Per the one-standard error method (Tibshirani et al., 2001), the optimal number of clusters is the smallest k such that $\mathrm{Gap}(k) \geq \mathrm{Gap}(k+1) - \mathrm{se}_{k+1}$. For the domains clustering, $k = 4$; for the symptoms clustering, $k = 6$. The gap statistic for the optimal k and the comparison to $k + 1$ are marked with dotted lines.

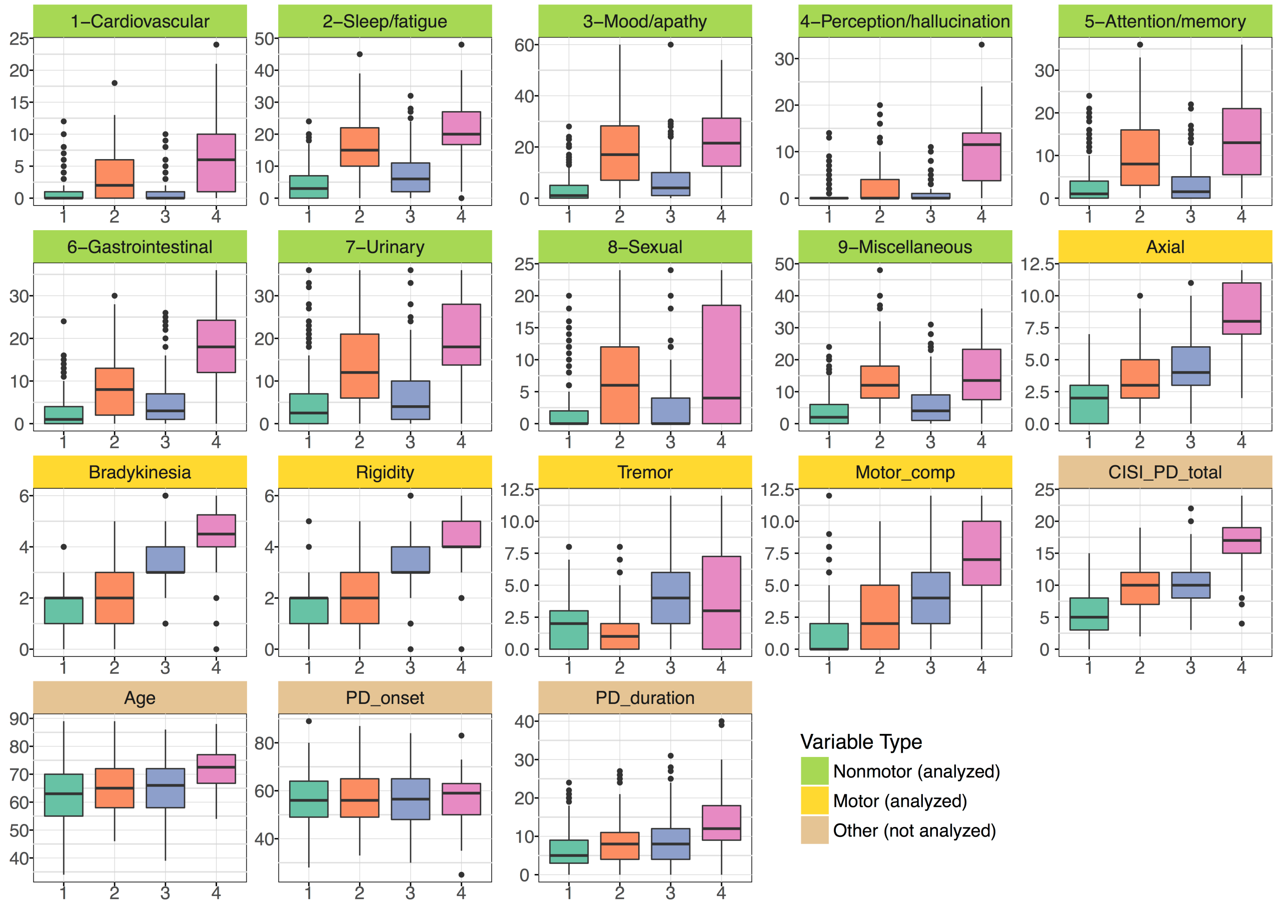

Supplementary Figure 2. Boxplots for domains clustering for each variable and cluster. This is an additional visualization of the data presented in Table 2 and Figure 1.

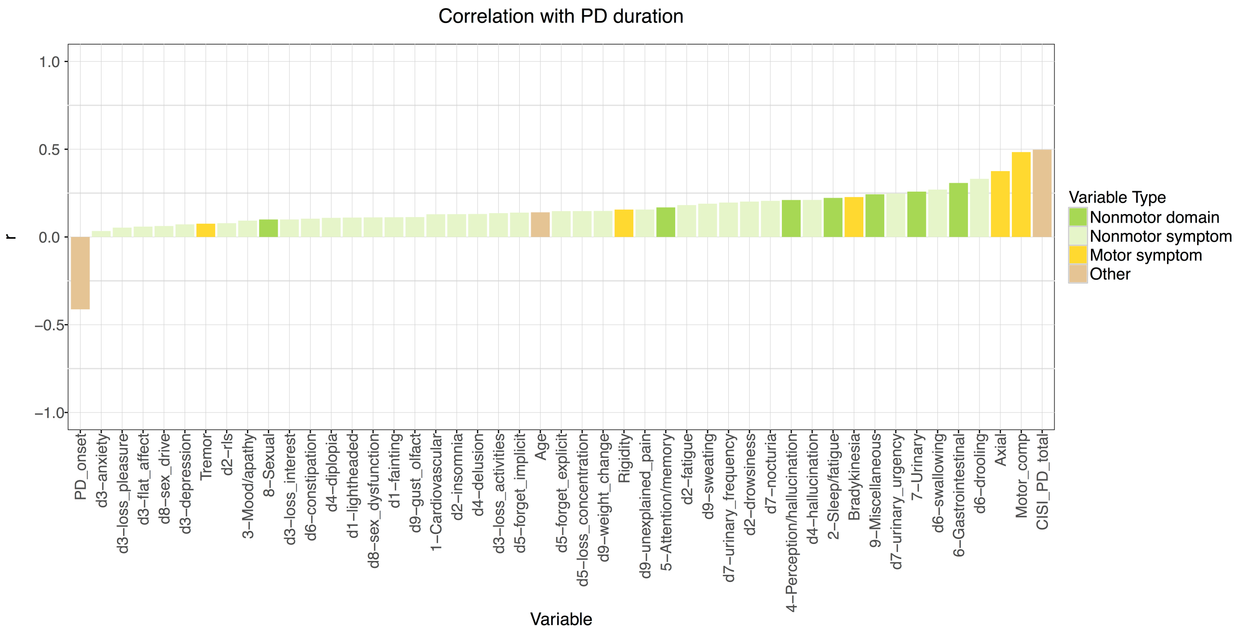

Supplementary Figure 3. Correlation of applicable variables with disease duration.
